# Resection of hypertrophic papillary muscles and mitral valve replacement in a patient with midventricular hypertrophic obstructive cardiomyopathy – a new approach

**DOI:** 10.1186/s13019-024-02529-w

**Published:** 2024-02-22

**Authors:** Julian J. Berg, Jan Eckstein, Marcus-André Deutsch, Jan F. Gummert, Masatoshi Hata

**Affiliations:** 1 Department of Thoracic and Cardiovascular Surgery, Heart and Diabetes Center North Rhine- Westphalia, University Hospital Ruhr-University Bochum, Bad Oeynhausen, Germany; 2 Institute of Radiology, Nuclear Medicine and Molecular Imaging, Heart and Diabetes Center North Rhine-Westphalia, University Hospital Ruhr-University Bochum, Bad Oeynhausen, Germany; 3 Department of Cardiovascular surgery, Osaka Police Hospital, Osaka, Japan

**Keywords:** Hypertrophic obstructive cardiomyopathy, Papillary muscles, Myectomy

## Abstract

Midventricular hypertrophic obstructive cardiomyopathy (HOCM) is characterized by hypertrophy of the interventricular septum (IVS) and - in rare cases - of the papillary muscles (PM), which subsequently can cause dynamic left ventricular outflow tract obstruction (LVOTO) and severe heart failure symptoms. We report on a rare case of a 44-year-old patient suffering from midventricular HOCM with hypertrophic anterolateral PM and an additional chorda between the PM and the IVS.

We describe a new surgical approach via right anterolateral thoracotomy in 3-dimensional (3D) video-assisted minimal-invasive technique with resection of hypertrophic PMs as well as the entire mitral valve-apparatus and valve replacement avoiding septal myectomy and potentially associated complications. After an uneventful procedure clinical symptoms improved from NYHA III-IV at baseline to NYHA 0-I postoperatively and remained stable over a follow-up period of 24 months. Therefore, the presented technique may be considered as a new and alternative approach in patients with hypertrophic PMs and hypertrophic IVS as subtype of midventricular HOCM.

## Background

Midventricular hypertrophic obstructive cardiomyopathy (HOCM) is a subtype of hypertrophic cardiomyopathy, which is characterized by hypertrophy of anterolateral and posteromedial papillary muscles (PM) and the interventricular septum (IVS). Additionally, anomalies of PMs and abnormal chordal attachment of the anterior mitral leaflet (AML) to the IVS can cause systolic anterior motion (SAM) and dynamic left ventricular outflow tract obstruction (LVOTO) with concomitant mitral regurgitation. Common treatment for HOCM is a myectomy of the hypertrophic IVS. However, surgical treatment of midventricular HOCM is usually challenging. The hypertrophic area cannot be reached via a transaortic approach. For that reason, a transapical ventriculostomy has been described as preferred access for surgical correction [1, 2]. In some cases of diffuse HOCM myectomy has been performed via trans-mitral septal myectomy with a video-assisted minimal invasive 2-dimensional (2D) technique [3].

We present a new approach with resection of hypertrophic PMs and MV replacement without septal myectomy in a patient with midventricular HOCM and hypertrophic PMs conducted via right anterolateral thoracotomy in 3-dimensional (3D) video-assisted minimal-invasive technique.

## Case presentation

**Fig. 1:**
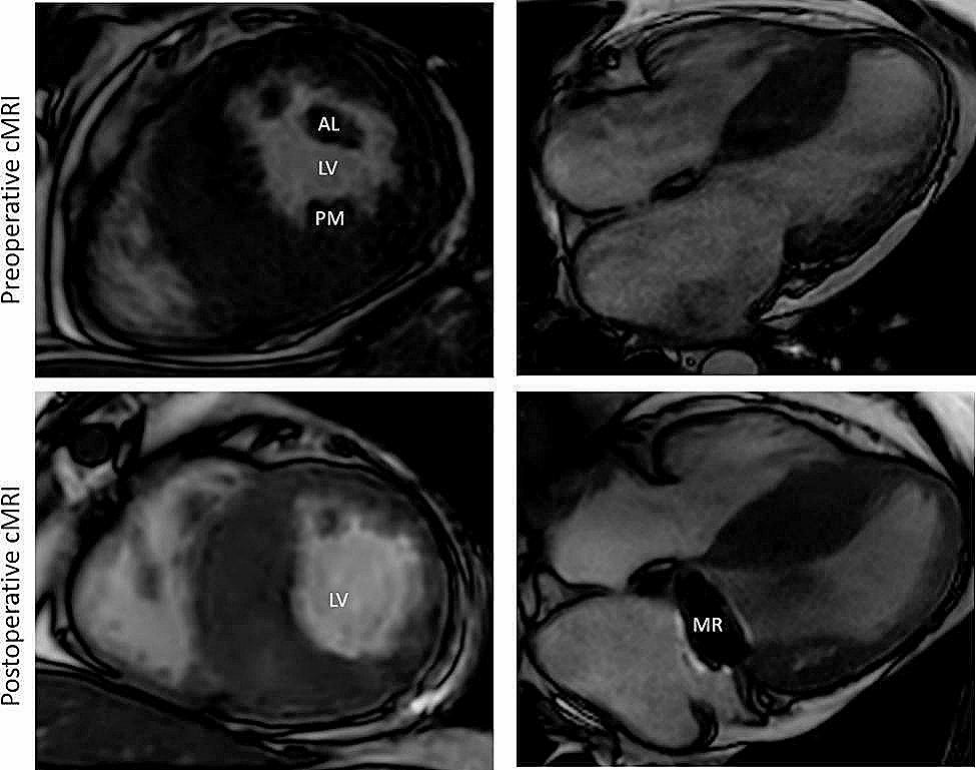
Short axis and 4-chamber view pre- and postoperative cMRI of the hypertrophic obstructive cardiomyopathy. The volume of the LV-cavity after resection of PMs increased. cMRI- cardiac magnetic resonance imaging, AL – anterolateral papillary muscle, PM – posteromedial papillary muscle, MR – mitral valve replacement

A 44-year-old man with known midventricular HOCM was referred to our center with progressive heart failure symptoms (NYHA III-IV) and atrial fibrillation, despite optimal medical therapy. Transthoracic echocardiography (TTE) revealed severe diastolic dysfunction of the left ventricle with an E-wave-amplitude of 147 cm/s, an E/e’ of 18,5 and a left atrium volume (LAV)-index of 65,4 ml/m^2^. It also showed a pronounced hypertrophic IVS causing a SAM phenomenon with concomitant mild mitral regurgitation and a peak left ventricular outflow tract (LVOT) gradient of 28mmHg with no aggravation during stress echocardiography. Transesophageal echocardiography (TEE) demonstrated significant hypertrophy of the anterolateral PM and an additional chorda between the PM and the IVS. To clarify the exact anatomy of involved structures cardiac magnetic resonance imaging (cMRI) was performed (Fig. 1* preoperative cMRI*). It showed a maximum IVS diameter of 29 mm at midventricular inferoseptal areas and confirmed PM hypertrophy and mild mitral regurgitation. Exercise stress electrocardiography showed horizontal ST-segment depressions in II, III and aVF with 1,3 − 1,5mV at J80-point with no T abnormalities. The septal branch appeared to be not suitable for alcohol ablation in cardiac angiogram.

**Fig. 2:**
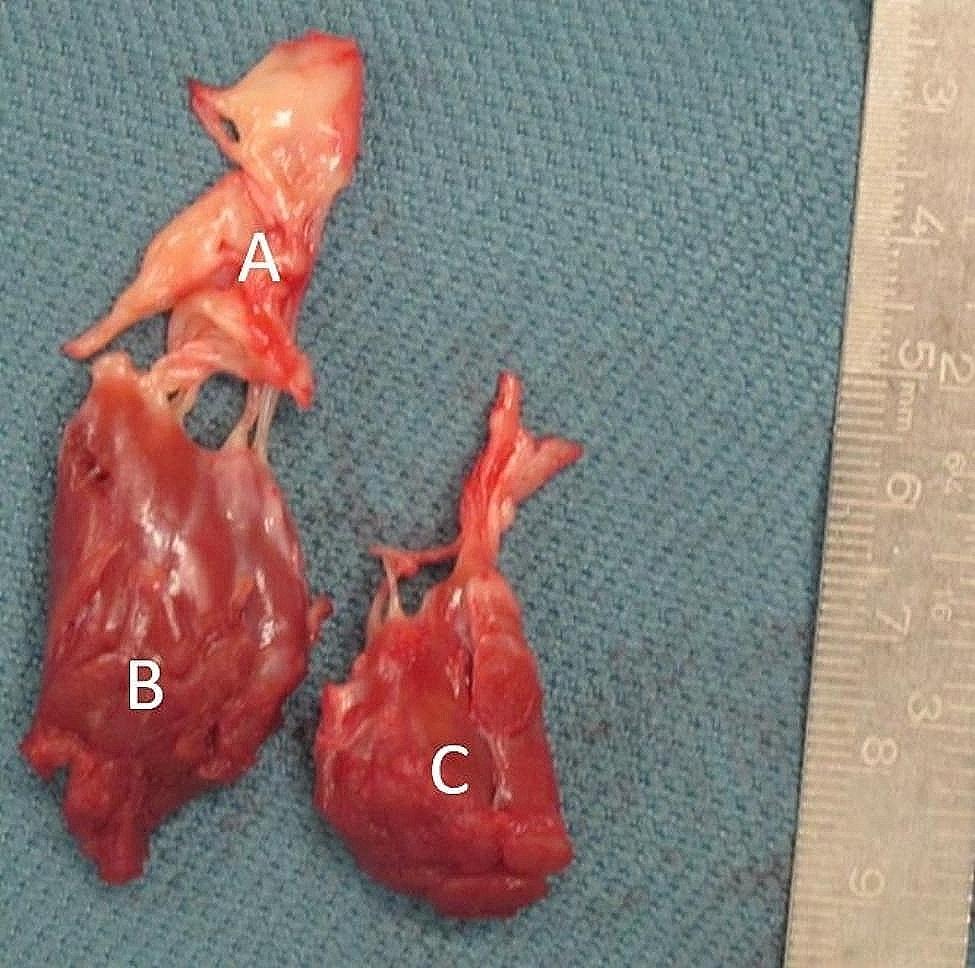
Resected MV apparatus. **A** Hypertrophic anterior Mitral leaflet, **B** anterolateral PM, **C** posterolateral PM

After interdisciplinary heart team discussion surgical treatment was recommended. We performed a right anterolateral mini-thoracotomy after initiating extracorporeal circulation via the femoral vessels. After aortic x-clamping and induction of cardioplegic arrest, the left atrium was opened. In order to obtain optimal view on subvalvular structures and to identify the hypertrophic structures a 3D-Video-system (Endoeye 3D, Olympus Co., Tokyo, Japan) was used. After cryoablation the MV apparatus including chordae tendineae and both hypertrophic PMs was resected (Fig. 2) and a mechanical valve prothesis (St. Jude Medical 31 mm) was implanted. Notably, myectomy of the IVS was not performed. Figure 1*postoperative cMRI* shows the postoperative result. Comparison between pre- and postoperative MRI (Table 1) found increased indexed LV enddiastolic volume from 45 ml/m² to 49 ml/m² with corresponding increase in LV stroke volume elevation from 79 ml to 84 ml respectively (Fig. 1*postoperative cMRI*).

**Table 1:** Pre- and postoperative MRI quantified cardiac functional parameters. LVEDV- left ventricular enddiastolic volume, LVEDVi- indexed left ventricular enddiastolic volume, LVESVi - indexed left ventricular endsystolic volume, LVEF – left ventricular ejection fraction, SV – stroke volume, HR – heart rate

Parameters	Preoperative MRI	Postoperative MRI
**LVEDV ml**	103	112
**LVEDVi ml/m²**	45	49
**LVESVi ml/m²**	19	25
**LVEF %**	65	70
**SV ml**	79	84
**HR bpm**	72	72

The postoperative course was uneventful and the patient was discharged without any residual symptoms. Two years after the operation, the patient remains free from symptoms.

## Discussion and conclusion

Midventricular HOCM with severe hypertrophy of PMs is a uncommon subtype of HCM, which can cause dynamic intraventricular obstruction without LVOTO [4]. In our case, TTE showed peak LVOT gradient of only 28mmHg but confirmed severe diastolic dysfunction of the LV with significant hypertrophy of PMs and IVS. The patient suffered from severe heart failure symptoms. This procedure was performed as last remaining option before being listed for heart transplantation.

Despite substantive myectomy, cMRI LV functional parameters only changed minimally, however clinical symptoms alleviated significantly from NYHA III-IV to NYHA 0-I and remained stable 24 months postoperatively. We consider that reduction of midventricular stenosis as shown by cMRI and reduction of diastolic LV dysfunction are the causes of clinical improvement. However, myectomy is reported to improve diastolic function in patients with HOCM [5].

Resection of the hypertrophic IVS in midventricular HOCM is usually challenging as mentioned above. Septal myectomy holds the risk of an iatrogenic ventricular septal defect (VSD) [6]. In comparison with IVS resection, the presented technique was simple, secure and effective avoiding sternotomy. However, to exclude any chance of SAM phenomenon the MV was replaced. 3D scope was helpful to resect papillary muscles precisely up to the level of the apex. Although we did not try additional cryoablation might be effective in this setting.

In conclusion, the presented technique may be considered as new and alternative approach in patients with hypertrophic PMs and hypertrophic IVS as subtype of midventricular HOCM.

## Data Availability

The datasets used and/or analyzed during the current study are available from the corresponding author on reasonable request.

## Declarations

### Authors’ information

MH has performed the operation.

## Abbreviations

AML: anterior mitral leaflet
HOCM: hypertrophic obstructive cardiomyopathy
IVS: interventricular septum
LAV: left atrium volume
LV: left ventricle
LVOT: left ventricular outflow tract
LVOTO: left ventricular outflow tract obstruction
MV: mitral valve
cMRI: cardiac magnetic resonance imaging
PM: papillary muscle
SAM: systolic anterior motion
TTE: transthoracic echocardiography
TEE: transesophageal echocardiography
VSD: ventricular septal defect
